# Association between BMP15 Gene Polymorphism and Reproduction Traits and Its Tissues Expression Characteristics in Chicken

**DOI:** 10.1371/journal.pone.0143298

**Published:** 2015-11-17

**Authors:** Haixia Han, Qiuxia Lei, Yan Zhou, Jinbo Gao, Wei Liu, Fuwei Li, Qian Zhang, Yan Lu, Dingguo Cao

**Affiliations:** 1 Institute of Poultry Science, Academy of Agricultural Sciences of Shandong Province, Jinan 250023, Shandong, China; 2 Poultry Breeding Engineering Technology Center of Shandong Province, Jinan 250023, Shandong, China; Zhejiang University, CHINA

## Abstract

BMP15 (Bone morphogenetic protein 15) is an oocyte-secreted growth factor required for ovarian follicle development and ovulation in mammals, but its effects on reproduction in chickens are unclear. In this study, the association between BMP15 polymorphisms and reproduction traits were analyzed, and its expression characteristics in different tissues were explored in LaiWu Black chickens. Three single nucleotide polymorphisms (SNPs) were identified in four hundred LaiWu Black chickens. One SNP (NC_006091.3:g.1773T>C) located in exon 2 which was significantly associated with egg weight at first egg (EWFE) (P = 0.0389), was novel. Diplotypes based on the three SNPs were found to be significantly associated with egg weight at age of 43W (EW43) (P = 0.0058). The chickens with H3H3 diplotype had their first egg 0.57 days later than chickens with H5H5 diplotype and 1.21 days-3.96 days earlier than the other five diplotype chickens. The egg production at age of 43W (E43), egg production at age of 46W (E46) and egg production at age of 48W (E48) for chickens with H3H3 diplotype were the highest among all the chickens, and the E48 of chickens with H3H3 diplotype had 11.83 eggs higher than chickens with H1H5 diplotype. RT-qPCR results showed that the expression level of *BMP15* gene in ovarian follicle was in the order of 4 mm>6 mm -8 mm> 15 mm -19 mm> 23 mm -29 mm > 33 mm -34 mm in diameter. The mRNA level in follicles of 4 mm and 6–8 mm in diameter were significantly higher than that in the other follicles (*P*<0.01). In the same week, the highest mRNA level was found in the ovary, and it was significantly different from that found in the liver and oviduct (*P*<0.01). Our results indicate that *BMP15* plays a vital role in the development of ovary and follicles, especially in the development of primary follicles. H3H3 may be an potential advantageous molecular marker for improving reproduction traits in chickens.

## Introduction

Bone morphogenetic protein 15 (BMP15) is a member of bone morphogenetic proteins (BMPs), which are multi-functional growth factors that belong to the transforming growth factor beta (TGF-beta) superfamily [1,2]. BMPs play critical roles in heart, neural, cartilage development, bone formation, and ovarian follicle development [3,4,5].

There is extensive evidence that BMP15 regulates granulosa cells proliferation and differentiation, ovarian folliculogenesis and appears to be crucial for female reproduction in mammals [6–9]. Mouse BMP15 is specifically expressed in the oocyte, beginning at the one-layer primary follicle stage and continuing through ovulation [10]. Knockout mouse technology also confirms the function of BMP15. BMP15 knockout female mice (Bmp15(-/-)) are sub fertile with decreased ovulation and fertilization rates [11]. Immature transgenic mice that overexpress BMP-15 exhibited accelerated follicle growth with decreased primary follicles and an increase in secondary follicles. Adult mice had normal litter sizes but an increased number of atretic antral follicles [12]. BMP15 is also found to be a pivotal factor inducing cumulus cell expansion [13]. BMP15 stimulates the expression of EGF-like growth factors in mouse cumulus cells as well as series of molecules downstream of EGF-like growth factor signaling, which are necessary for normal cumulus expansion [14]. BMP15 expression pattern is associated specifically with the period of cumulus cell expansion during in vitro maturation of buffalo [15]. The most important function of BMP15 for ovarian follicular development in sheep [16], zebrafish [17], cattle [18], human [19] and pig [20,21] have also been confirmed. Untill now, eight genetic mutations with major effects on ovulation rate and litter size have been identified in BMP15. They are FecX^I^ in Inverdale sheep, FecX^B^ in Belclare sheep, FecX^G^ in Cambridge sheep, FecX^H^ in Hanna sheep, FecX^L^ in Lacaune sheep, FecX^R^ in Rasa Aragonesa sheep and FecX^Gr^ FecX° in the Grivette and Olkuska sheep. However, these mutations are not applicable to all breeds of sheep [22–27].

In chicken, BMP15 is found to be preferentially expressed in the ovary, low in the brain, but not found in other tissues. The BMP15 expression was maintained during hierarchical follicular maturation in the germinal disc region and then progressively decreased after ovulation [28]. Despite identifying BMP15 biological functions in mammals and other animals, its biological functions in chicken are still unclear. Also, nothing is known about its genetic mutations in chicken. Hence, this study was designed to analyze the expression characteristics of BMP15 gene in different tissues, discover the potential molecular genetic markers that are related to reproduction traits in LaiWu Black chicken, and explore the biological effect of BMP15 on chicken reproduction traits.

## Materials and Methods

### Ethics Statement

All experiments were approved by the Animal Care Committee of the Academy of Agricultural Sciences, Shandong Province, Ji’nan, China. The care and use of experimental animals were carried out in accordance with the Directory Proposals on the Ethical Treatment of the Experimental Animals, established by the Ministry of Science and Technology (Beijing, China).

### Animals and Reproduction traits

Four hundred LaiWu Black chickens were used for the detection of SNPs in BMP15 and the analysis of the relationship between the SNPs and the reproduction traits. All the birds were selected randomly from the conservation population maintained by Local Breed Genetic Resources Bank of Shandong Province. All birds were hatched on the same day, reared in a stair-step cage under the same nutritional and environmental conditions, and transferred later to the single cage at the age of 100 d. Six reproduction traits were measured according to The Poultry Production Performance Terms and Measurement Statistics Method (NY/T823-2004). These traits include age at first egg (AFE), egg weight at first egg (EWFE), egg weight at age of 43W (EW43), egg production at age of 43W (E43), egg production at age of 46W (E46) and egg production at age of 48W (E48). Venous blood samples were collected from all four hundred LaiWu Black chickens by venipuncture. The genomic DNA was isolated with the TIANamp Blood DNA Kit (DP318, Tiangen, Beijing, China) according to the manufacturer’s instructions, and then stored at -20°C for genotype.

Five LaiWu Black chickens were randomly selected for the tissue sampling of the liver (L), oviduct (S) and ovary (O) at the age of 19W, 23W and 40W, respectively. Also, all levels of follicles were obtained at the age of 40W. The follicles were classified according to Onagbesan (2009) [29] as F1 (33mm-34mm diameter), F2/F3 (23mm-29mm diameter), F4/F5 (9mm-15mm diameter), yellow follicles (6mm-8mm diameter) and white follicles (4mm diameter). Total RNA was isolated from the tissues using Ultrapure RNA Kit (CW0581, CWbio.Co.Ltd, Beijing, China) according to the manufacturer’s instructions, and then stored at -80°C for tissue expression analysis.

### Detection of SNPs, Genotype, and Construction of haplotypes and diplotypes

Six DNA pools based on 180 samples were constructed to detect the genetic mutations of BMP15. Each pool was made up of 30 DNA samples in equal concentration (5 μL of genomic DNA (50ng/μL) for each sample). According to the CDS sequence of BMP15 (GenBank Accession no. NC_006091.3, GI: 358485508), two sets of PCR primers (BMP15P1 and BMP15P2, Table 1) were designed with the Primer Premier5.0 to amplify the DNA pool for the identification of SNPs in BMP15. The PCR was performed in 15 μL volume system containing 1 μL of genomic DNA (50ng/μL), 0.45 μL of each primer (10pmol/μL), 5.6 μL ddH_2_O, and 7.5 μL of 2×MasterMix (Tiangen, Beijing, China). The PCR cycle conditions used include one denaturation step at 94°C for 5 min; 35 cycles at 94°C for 30 s, 58.4°C or 59.4°C (depends on the primer pair used) for 30 s, and 72°C for 60s; and a final elongation at 72°C for 10 min. The PCR products were purified with AxyPrep^TM^ DNA Gel Extraction Kit (Axygen, Union City, CA, USA) and sequenced by Jinan Li Ge Technology Co., Ltd (Jinan, China). All sequences were analyzed with DNAMAN7.0 and Chromas 2.31 software.

**Table 1:** Primer parameters for SNPs identification and genotyping of *BMP15*.

Primer	Sequences (5′–3′)	Length of products/bp	Annealing temp (°C)	Usage
*BMP15*P1	F: GGGACCTCTTTCTGCTTTACCR: CATCACCCATTGCCACCA	406	58.4	SNPs identification
*BMP15*P2	F: GGACAACAAGGGCAAGGGR: ATCGCATCGAGTGGAGACAA	1096	59.4	SNPs identification
NC_006091.3:g.474A>G	F: GGGACCTCTTTCTGCTTTACCR: CATCACCCATTGCCACCA	406	58.4	Genotyping with *Hha*I
NC_006091.3:g.594C>T	F: GGGTTTTAGCCCTGATCTTGCACTCR: ATCACCCATTGCCACCACCTTACCT	517	58	Genotyping with *Alw21*I
NC_006091.3:g.1773T>C	F: TGAGCACCTTCTCCGTGTCAR: ATCCAATGGTCCCAACCC	462	59.6	Genotyping with *Mva*I

Then, three genetic mutations (NC_006091.3:g.474A>G, NC_006091.3:g.594C>T and NC_006091.3:g.1773T>C) were found and genotyped by PCR-RFLP with primers (Table 1). The primer of g.594C>T was referenced in Li et al. (2012) [30]. The PCR were performed as described above. PCR-RFLP reactions were performed in a 15 μL volume system containing 0.5 μL of restriction enzymes (10U/μL) (*Hha*I, *Alw21*I or *Mva*I, New England Biolabs, Inc., USA), 1 μL of 10× NEBuffer, 8 μL of PCR productions and 5.5 μL ddH_2_O. The PCR products were digested at 37°C or 65°C for 4 h, and then the digested products were separated on a 2.5% agarose gel for 30 min at 120V. After the genotyping, two samples for each genotype of each SNP were sequenced by Jinan Li Ge Technology Co., Ltd (Jinan, China) to confirm the variation. Haplotypes and diplotypes were constructed based on the SNPs identified in all 400 birds using the PHASE 2.0 software.

### Real-time quantitative PCR

The total RNA isolated from tissues was assessed using the A260/A280nm ratio with the expected values falling between 1.8 and 2.0 (Eppendorf, Hamburg, Germany). 1μg of total RNA was used for cDNA synthesis with HiFi-MMLV cDNA Kit (CW0744, CWbio.Co.Ltd, Beijing, China) according to the manufacturer’s instructions.

The chicken *β-actin* gene was used as a control for RT-qPCR. According to the mRNA sequences of BMP15 (GenBank Accession no. NM_001006589.2, GI: 55742820) and *β-actin* (GenBank Accession no. NM_205518.1, GI: 45382926), the primers for real-time PCR (Table 2) were designed with the Primer Premier5.0. The real-time quantitative PCR reactions were conducted with Roche LC-480II (Roche, California, USA) in a 20μL-volume system containing 10 μL UltraSYBR Mixture (with Rox) (CW0956, CWbio.Co.Ltd, Beijing, China), 0.4 μL of each primer (10pmol/μL), 2 μL cDNA, and 7.2 μL ddH_2_O. The PCR cycle condition is as follows: one denaturation step at 95°C for 10 min, followed by 40 cycles of 95°C for 15 s and 60°C for 60 s. The specificity of the PCR productions was checked by a final melting curve analysis. The cycle threshold value of control gene was used to normalize the target gene signals in each sample. Standard curves were generated for each gene by serial dilution to quantify the amplified products. Each sample was amplified in triplicate, and the mean value of each triplicate was used for further analysis. The 2^-△△CT^ method was used to calculate the relative expression of transcripts between the target gene and the control gene.

**Table 2:** Primer parameters for RT-qPCR.

Gene	Primer sequence(5’-3’)	Annealing temp (°C)	Product size(bp)
*BMP15*	F: TTGATGCTTGGTGGGTGGTTR: CACCATAGACTGCCTCGTTC	60	177
*β-actin*	F: CCATCTATGAAGGCTACGCR: CTCGGCTGTGGTGGTGAA	60	124

### Statistical analysis

The Hardy-Weinberg equilibrium was evaluated by an χ2 test. The association between SNPs or diplotypes with reproduction traits were analyzed by the GLM procedures of SAS8.12 (SAS Inst. Inc., Cary NC, USA), and the estimated genotypes values were compared by Duncan’s Multiple Range Test (SAS8.12).The statistical model was shown as following:

$$\text{Y}=\mu +{\text{G}}_{\text{i}}+\text{e}$$

Where Y = the phenotypic value of traits, μ = the population mean, G = fixed effects of genotype or diplotype, and e = random residual error. Multiple comparisons were performed with the least squares means. One-way ANOVA was used to examine the mRNA expression differences among different tissues in chicken. All the values were considered significant at *P* < 0.05 and are presented as least square means ± standard error means.

## Results

### Genotype, allele frequencies, and association analysis

The three genetic mutations (NC_006091.3:g.474A>G, NC_006091.3:g.594C>T and NC_006091.3:g.1773T>C) were genotyped by PCR-RFLP, and a total of eight genotypes were detected. By sequencing, the nucleotide variation at each locus was further confirmed. Relative to GenBank Accession No. NC_006091.3, an A→G mutation at position 474 nucleotide (NC_006091.3:g.474A>G) and a C→T mutation at position 594 nucleotide (NC_006091.3:g.594C>T) were located on exon 1, and a T→C mutation at position 1773 nucleotide (NC_006091.3:g.1773T>C) was located on exon 2. All the three SNPs did not cause amino acid change.

Genotypes and alleles frequency analysis showed that (Table 3) in NC_006091.3:g.474A>G, A was the advantageous allele. In NC_006091.3:g.594C>T, the frequency of CT was higher than that of the CC and TT genotypes, and allele C was dominant. In NC_006091.3:g.1773T>C, C was the dominant allele since the CC genotype (0.957) occurred much more frequently than the other genotypes. The genotype distributions for all the three SNPs fit the Hardy-Weinberg equilibrium with the P-value higher than 0.05.

**Table 3:** Genotypes and alleles frequencies at site NC_006091.3: g.474A>G, NC_006091.3:g.594C>T and NC_006091.3:g.1773T>C of *BMP15* gene.

SNPs	Location	Genotypes frequency			Allele frequency		*P-value* ^a^
NC_006091.3:g.474A>G	Exon1	AA	AG	GG	A	G	
		0.742	0.235	0.023	0.860	0.140	0.6299
NC_006091.3:g.594C>T	Exon1	CC	CT	TT	C	T	
		0.265	0.505	0.230	0.518	0.482	0.8221
NC_006091.3:g.1773T>C	Exon2	TT	TC	CC	T	C	
		0.000	0.043	0.957	0.021	0.979	0.6641

^*a*^ *P-value* is the probability of the χ2-text for the Hardy-Weinberg equilibrium

The association between genotypes and six reproduction traits were estimated with a total of 398 chickens because traits recording of two chickens went missing. The results are summarized in Table 4. For NC_006091.3:g.474A>G, the association was not significant with EW43 although a trend was observed (P = 0.0519). For NC_006091.3:g.594C>T, the genotypes had no significant assocaitons with AFE (P = 0.0534), EW43 (P = 0.0529) and E48 (P = 0.0683). For NC_006091.3:g.1773T>C, there was a significant association with EWFE (P = 0.0389). The AFE, EW43, E46 and E48 of chickens with N1N2 genotype were superior to chickens with N2N2 genotype, but no significant difference was observed between them (P>0.05).

**Table 4:** Least-squares means and standard errors for reproduction traits of different genotypes in SNPs.

Traits	P -Value	NC_006091.3:g.474A>G		
		H1H1 (295)	H1H2 (94)	H2H2(9)
EWFE(g)	0.9359	30.47±0.25	30.29±0.44	30.22±1.45
AFE	0.6978	139.55±0.52	140.02±0.93	137.44±3.01
EW43(g)	0.0519	47.62±0.21^a^	47.47±0.38^a^	44.34±1.32^b^
E43	0.8166	117.85±1.25	116.78±2.20	114.00±7.44
E46	0.7993	130.82±1.38	129.83±2.43	125.87±8.21
E48	0.7956	139.19±1.47	137.81±2.59	134.50±8.74
		NC_006091.3:g.594C>T		
		M1M1(105)	M1M2(201)	M2M2 (92)
EWFE (g)	0.9881	30.37±0.42	30.45±0.30	30.42±0.45
AFE	0.0534	140.99±0.87^a^	139.69±0.63^a^	137.88±0.93^b^
EW43(g)	0.0529	47.76±0.36^a^	47.76±0.25^a^	46.66±0.39^b^
E43	0.1772	114.60±2.06	117.87±1.50	120.25±2.28
E46	0.1352	127.21±2.27^b^	130.70±1.66^ab^	134.00±2.52^a^
E48	0.0683	134.80±2.42^b^	138.95±1.76^ab^	143.19±2.68^a^
		NC_006091.3:g.1773T>C		
		N1N2(17)	N2N2(381)	
EWFE (g)	0.0389*	28.29±1.05^b^	30.51±0.22^a^	
AFE	0.4846	138.11±2.19	139.68±0.46	
EW43(g)	0.2865	46.63±0.85	47.56±0.19	
E43	0.8110	118.70±5.10	117.45±1.10	
E46	0.7426	132.29±5.62	130.40±1.21	
E48	0.5950	141.88±5.99	138.61±1.29	

The least square means within a row lacking a common lowercase superscript differ significantly (*P*<0.05).

The numbers in the brackets are the chicken individuals of respective genotypes.

### Construction of haplotypes and association analysis

The parameters of haplotypes and diplotypes based on the three SNPs are shown in Table 5. A total of six haplotypes were obtained, and A-T-C, A-C-C, and G-C-C were the main haplotypes, accounting for 97.625% of the observations. Eleven diplotypes were obtained based on the six haplotypes. To ensure that the analysis was accurate, four haplotypes with a frequency lower than 2% were not used in the further association analysis.

**Table 5:** Haplotypes and diplotypes inferred based on the 3single nucleotide polymorphisms.

Haplotype	NC_006091.3:g.474A>G	NC_006091.3:g.594C>T	NC_006091.3:g.1773T>C	Frequency (%)	Diplotype	Frequency(%)	Diplotype	Frequency(%)
H1	A	C	C	36.125	H1H1	12.750	H3H5	12.500
H2	A	C	T	2.000	H1H2	1.000	H3H6	0.250
H3	A	T	C	47.750	H1H3	36.000	H4H6	0.250
H4	A	T	T	0.125	H1H5	9.750	H5H5	2.250
H5	G	C	C	13.750	H2H3	2.250		
H6	G	T	C	0.250	H2H5	0.750		
					H3H3	22.250		

The least squares mean multiple comparisons of diplotypes are shown in Table 6. Diplotypes was found to be highly significantly associated with EW43 (P = 0.0058). With the increase in chickens’ age, the association between the diplotypes and egg production increased. The AFE of chickens with H3H3 diplotype was 138.01 days old, which was 0.57 days later than chickens with H5H5 diplotype but 1.21 days-3.96 days earlier than the other five diplotype chickens. Meanwhile, the E43, E46 and E48 of chickens with H3H3 diplotype were the highest among all the chickens, and the E48 of chickens with H3H3 diplotype had 11.83 eggs higher than that of chickens with H1H5 diplotype. The H1H5 chickens had the highest AFE and lowest E43, E46, E48 among the other chickens.

**Table 6:** Association of diplotypes of chicken *BMP15* gene with the reproductive traits.

Traits	EWFE (g)	AFE	EW43 (g)	E43	E46	E48
P value	0.8798	0.2606	0.0058**	0.4918	0.3473	0.1953
H1H1(50)	**30.98±0.61** ^1^	141.26±1.27	**48.57±0.52** ^1^	114.98±2.98	127.90±3.28	135.50±3.48
H1H3(143)	30.50±0.36	139.82±0.75	47.97±0.30	117.13±1.80	129.56±1.98	137.62±2.10
H1H5(39)	30.07±0.70	**141.97±1.44** ^1^	47.59±0.59	*112*.*71±3*.*42* ^2^	*124*.*86±3*.*76* ^2^	*131*.*78±4*.*00* ^2^
H2H3(9)	*28*.*77±1*.*45* ^2^	139.22±3.00	46.04±1.15	118.00±7.02	131.77±7.73	141.22±8.21
H3H3(89)	30.43±0.46	138.01±0.95	46.62±0.40	**120.74±2.34** ^1^	**134.48±2.57** ^1^	**143.61±2.73** ^1^
H3H5(50)	30.58±0.61	139.40±1.27	47.33±0.51	119.85±3.04	133.77±3.34	142.43±3.55
H5H5(9)	30.22±1.45	*137*.*44±3*.*00* ^2^	*44*.*34±1*.*31* ^2^	114.00±7.45	125.87±8.20	134.50±8.71

^1^ Bold values represent the advantageous diplotypes

^2^ Italic values represent the negative diplotypes.

** *P* ≤ 0.01

### Tissue expression analysis of BMP15

**Fig 1:**
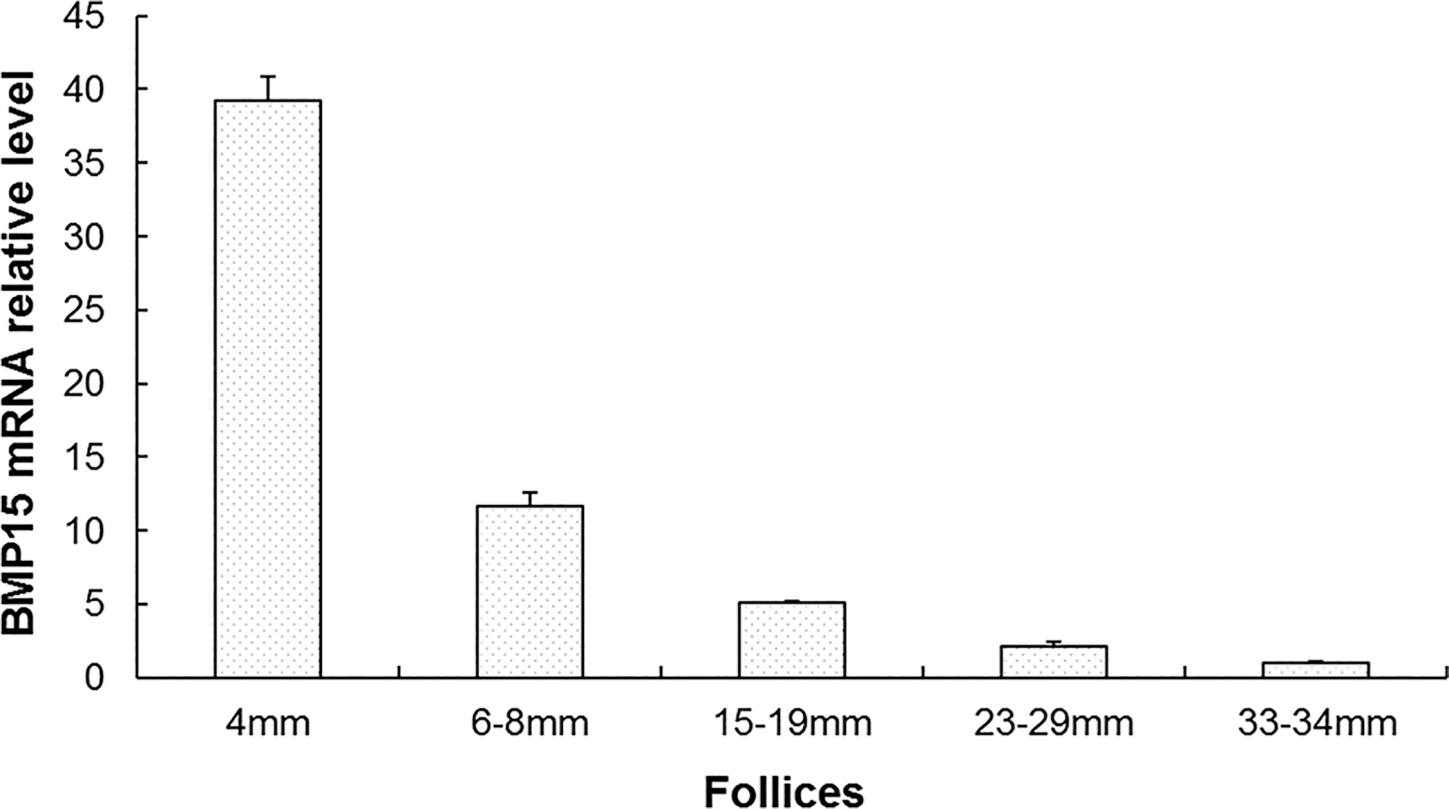
Relative expression level of *BMP15* mRNA in follicles of 40w Laiwu Black chicken.

The mRNA relative expression of BMP15 in different levels of follicles is shown in Fig 1. The expression level of *BMP15* in ovarian follicle was in the order of 4 mm>6 mm -8 mm> 15 mm -19 mm> 23 mm -29 mm > 33 mm -34 mm in diameter. The mRNA level of *BMP15* in follicles with a diameter of 4 mm and 6–8 mm were significantly higher than that in the other follicles (*P*<0.01).

**Fig 2:**
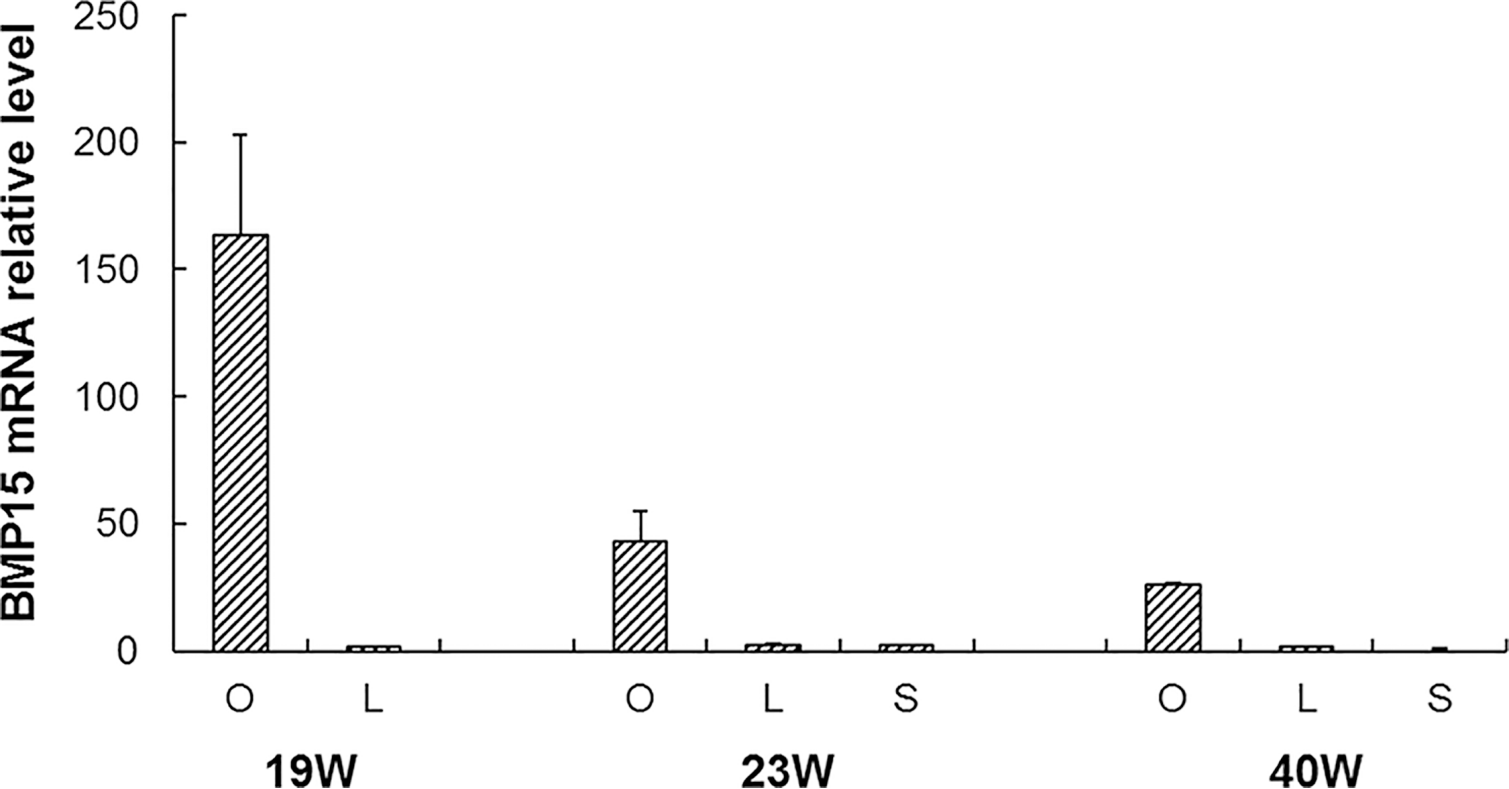
The expression of a chicken *BMP15* gene in liver, ovary and oviduct at the age of the same week.

The differential expression pattern of BMP15 in different tissues in the same week is presented in Fig 2. In the same week, the trends of the mRNA level in various tissues were ovary > oviduct > liver. The highest mRNA level was found in the ovary, and it was significantly different from that found in the liver and oviduct (*P*<0.01).

## Discussion

The oocyte factor, bone morphogenetic protein-15 (BMP-15) has proven to be critical for normal fertility in female mammals [31,32]. Wu (2009) detected a T→A mutation of exon 2; total number born and number born alive for BB pigs were significantly lower than AA and AB pigs for both Xiaomeishan pig and Large White pig (P<0.05) [33]. Li L et al. (2009) found two SNPs in rabbit BMP15, and the mean litter size of BB genotypes was higher than that of AA and AN genotype in angora rabbit flock (P<0.05) [34]. Shabir M, et al. (2013) found that SNP "C" of mutations in exon-2 of the designated genotype AC was observed to produce a significant effect on litter size with average litter size going up by 0.63, as compared with the nearest genotype AB with the litter size of 1.29±0.05 [35]. In sheep, eight genetic mutations with major effects on ovulation rate and litter size have been identified in BMP15 [22–27]. In Shaobo hens, Huang HY, et al. (2015) detected three SNPs in each of BMP15 (A111G, C231T and C34T) and GDF9 (G593A, T824C and C896T), and found that the C34T had an effect on total egg production at 300 d of age (EN) and age at first laying (AFE), G593A affected EN and both C231T and C896T influenced AFE [36]. But our results showed that only NC_006091.3:g.594C>T had a trend of association with AFE (P = 0.0534), EW43 (P = 0.0529) and E48 (P = 0.0683), but the association was not significant (P>0.05). The difference between results may be caused by the different experimtal chicken populations we used, and the different SNPs we detected. The C34T they detected leads to the substitution of Leu by Phe, which was predicted to affect protein function. But In this study, all the three SNPs (NC_006091.3:g.474A>G and NC_006091.3:g.594C>T and NC_006091.3:g.1773T>C) were synonymous mutations. NC_006091.3:g.1773T>C was significantly associated with EWFE (P = 0.0389). Synonymous mutations do not change the sequence or structure of the protein, once was thought to be functionally neutral, but evidence now indicates it is shaped by evolutionary selection and affects other aspects of protein biogenesis beyond specifying the amino acid sequence of the protein [37]. Studies analyzing the consequences of synonymous codon changes in different organisms have revealed that they impact nucleic acid stability, protein levels, structure and function without altering amino acid sequence [38]. Furthermore, gene expression is correlated with synonymous codon usage bias [39,40]. Thus for the SNPs, the condon choice may affect the BMP15 gene expression or the coordinated expression of functionally related genes, further affect the follicle development, ovulation and the reproduction traits in chicken. However, much remains unknown about the molecular mechanisms connecting synonymous codon usage to efficient protein biogenesis and proper cell physiology [37]. So, the mechanism of the three SNPs of BMP15 to reproduction traits in chicken should be further studied.

Diplotypes was found to be significantly associated with EW43 (P = 0.0058), and the association between them increased with the increase in chickens’ age (in days). The AFE of H3H3 chickens was 138.01 days old, which was 0.57 days later than H5H5 chickens but 1.21 days-3.96 days earlier than the other five diplotype chickens. Meanwhile, the E43, E46 and E48 of H3H3 chickens were the highest among all the chickens, and the E48 of H3H3 was 11.83 eggs higher than that of the H1H5 chickens. So, the H3H3 was regarded as the advantageous diplotype for reproduction trait. The H1H5 chickens had the highest AFE and lowest E43, E46, E48 among the other chickens. As a result, it was regarded as the detrimental diplotype for chicken reproduction traits, suggesting that it should be deleted during the cultivation of new varieties. These findings showed that BMP15 has a significant effect on the reproduction traits in chicken, similar to its function in the other animals.

The function of BMP15 in chicken reproduction was further confirmed by analyzing the mRNA expression pattern. Bone morphogenetic protein 15 is a major and well-known oocyte-secreted growth factor, required for follicular development and ovulation [41,42]. Eckery et al. (2002) found that the BMP15 protein was observed in oocytes from the primordial stage of follicular formation and the results suggest a possible role for these proteins in the maintenance of primordial follicles, as well as playing a key role during follicular development in the brushtail possum [43]. Using real-time PCR, Paradis et al. (2009) revealed that BMP15 mRNA was most abundant in the oocyte; its expression remained relatively constant during follicular development in pig [44]. Sun (2010) found that BMP15 was presented in oocytes and granulosa cells of all follicles in mice and porcine [45]. Our results showed that *BMP15* mRNA was expressed in all ovarian follicles of Laiwu Black chickens, and strongly expressed in follicles with 4 mm and 6–8 mm diameter. Moreover, in contrast to oviduct and liver, BMP15 was preferentially expressed in the ovary during the same week, which was consistent with the findings of He X L, et al. (2009) [46], Wang Y, et al. (2012) [47] and Sudiman J et al. (2014) [48]. Collectively, the findings suggest that BMP15 might be involved in ovarian follicular growth and development, and plays a major role in primordial follicular recruitment in chicken.

In Conclusion, the expression level of *BMP15* was different in different tissues; the expression level in the ovary was higher than that in the other tissues. Also, the highest mRNA level was found in 4 mm follicles, indicating that *BMP15* played a vital function in the development of ovary and follicles, especially in the development of primary follicles. H3H3 could be used as a potential advantageous molecular marker for reproduction traits in chickens. This research provides exciting new opportunities for understanding the role of the oocyte-secreted factor BMP-15 on ovarian follicular growth and development in chicken.
